# Melatonin Alleviates Drought Stress in Sweet Sorghum Seedlings via Protection of Photosynthetic Apparatus and Carbon-Nitrogen Metabolism

**DOI:** 10.3390/ijms27125291

**Published:** 2026-06-11

**Authors:** Nuerkaimaier Mulati, Mengke Wang, Shangfu Ren, Ting Wang, Kun Zhang, Lu Li, Cuijie Cui, Li Yu, Liping Zhu

**Affiliations:** 1 College of Life and Geographic Sciences, Kashi University, Kashi 844000, China; 18899355590@163.com (N.M.); wang315047@163.com (M.W.); renshagnfu@163.com (S.R.); zhangkun_2022@163.com (K.Z.); 13919189679@163.com (L.L.); ccj19987654321@163.com (C.C.); 2 Key Laboratory of Biological Resources and Ecology of Pamirs Plateau in Xinjiang Uygur Autonomous Region, Kashi University, Kashi 844000, China; 3 College of Life Sciences, Xinjiang Agricultural University, Urumqi 830000, China; 4 National Key Laboratory of Cotton Bio-Breeding and Integrated Utilization, School of Life Science, Henan University, Kaifeng 475000, China

**Keywords:** *Sorghum bicolor* L., drought stress, melatonin, transcriptome

## Abstract

Sweet sorghum, a high-quality forage and energy crop, is significantly affected by drought, the primary abiotic stress impacting its growth. Melatonin (MT) has emerged as a crucial signaling molecule in plant responses to abiotic stress. This study investigates the role of melatonin in enhancing drought tolerance in sweet sorghum, specifically using the ‘Dali Shi’ variety under polyethylene glycol (PEG)-induced drought conditions. Our findings demonstrate that exogenous melatonin application significantly increased proline content (by 27.76% and 5.95% under mild and moderate drought, respectively) while decreasing malondialdehyde (MDA) levels (by 18.33% and 35.18%, respectively). Melatonin also enhanced the accumulation of photosynthetic pigments, including chlorophyll b and total chlorophyll, and improved photosynthetic fluorescence parameters (Fv/Fm and ETR). Additionally, melatonin treatment improved root vitality, stimulated carbon and nitrogen metabolism, and increased antioxidant enzyme activity. Transcriptomic analysis revealed that differentially expressed genes were enriched in pathways related to carbon fixation, glycolysis/gluconeogenesis, nitrogen metabolism, antioxidant defense, and plant hormone signaling. Notably, melatonin upregulated key genes associated with these pathways and activated bHLH and MYB transcription factors. In conclusion, this study elucidates the mechanisms by which melatonin enhances sweet sorghum’s drought tolerance, highlighting its potential as a practical approach for improving drought resistance in this crop.

## 1. Introduction

In China, drought is the most severe abiotic constraint on crop production, affecting more than half of the total agricultural area impacted by all environmental stresses and causing billions of dollars in economic losses annually [1]. Water availability is the primary factor determining crop yield in arid and semi-arid regions. Xinjiang, located in the northwest inland arid zone, faces chronic water shortages. The soils there are typically dry and infertile, leading to low land productivity. This situation not only restricts the cultivation of conventional crops but also exacerbates the conflict between forage production and limited water resources. Consequently, drought has become a key limiting factor for local livestock development [2].

*Sorghum bicolor* L. is a C_4_ crop characterized by rapid growth, high biomass yield, strong adaptability, and tolerance to both drought and salinity. These traits make it an ideal forage and energy crop for marginal lands and low-yield fields in northwestern China [3,4,5]. Although Xinjiang is located in an arid region with relatively low land quality, it benefits from abundant sunlight. Sorghum has been introduced as a new quality forage in Xinjiang, and the planting area of sorghum in the region has increased by 0.67 to 1.37 million hectares since 2016, significantly alleviating the forage pressure caused by cattle and sheep farming. Despite the strong adaptability of *Sorghum bicolor* to drought conditions, drought remains a primary factor affecting crop production [6,7].

Melatonin (MT) is an indole compound widely present in various organisms. It enhances plant tolerance to abiotic stresses such as drought by scavenging reactive oxygen species (ROS) and regulating the activity of antioxidant enzymes [8,9,10,11]. Exogenous application of melatonin has been shown to improve seed germination under drought stress and to enhance seedling adaptability [12,13]. Bai et al. reported that melatonin treatment promotes biomass accumulation and enriches nutrient reserves in cotton seeds, thereby improving germination and reducing yield losses [11]. Similarly, Chen demonstrated that melatonin enhances photosynthetic efficiency and modulates gene expression, which in turn optimizes plant growth and yield [14,15].

Additionally, melatonin promotes formation of lateral and adventitious roots, thereby increasing root vitality under stress conditions. Exogenous melatonin has been shown to stimulate root elongation in rapeseed [16] and tomato [17], enhancing water uptake and drought resistance. Liu et al. reported that melatonin facilitates adventitious root regeneration in tomato seedlings through receptor-dependent pathways [18]. In wheat, melatonin also exhibits drought resistance during seed germination and seedling stages. This effect is primarily achieved by regulating water metabolism, improving leaf water retention capacity, and promoting root development. Du et al. [19] discovered that melatonin can regulate the glycolytic pathway in celery. Specifically, it upregulates the expression of pyruvate synthesis genes (*AgPEP1* and *AgPK3*) while downregulating degradation genes (*AgPDC2* and *AgPDHA2*). This regulation promotes pyruvate accumulation, enhances peroxidase activity, and ultimately improves drought resistance in celery [14,19].

Despite these findings, the molecular and physiological mechanisms by which melatonin regulates drought resistance in sweet sorghum remain unclear. Therefore, this study aimed to elucidate how exogenous MT influences sweet sorghum seedlings under drought stress and to analyze the associated metabolic pathways. The results will provide valuable insights for using MT to enhance drought resistance in cereal production.

## 2. Results

### 2.1. The Effect of Exogenous MT on the Phenotypic Growth of Sweet Sorghum Seedlings Under PEG-Simulated Drought

**Figure 1:**
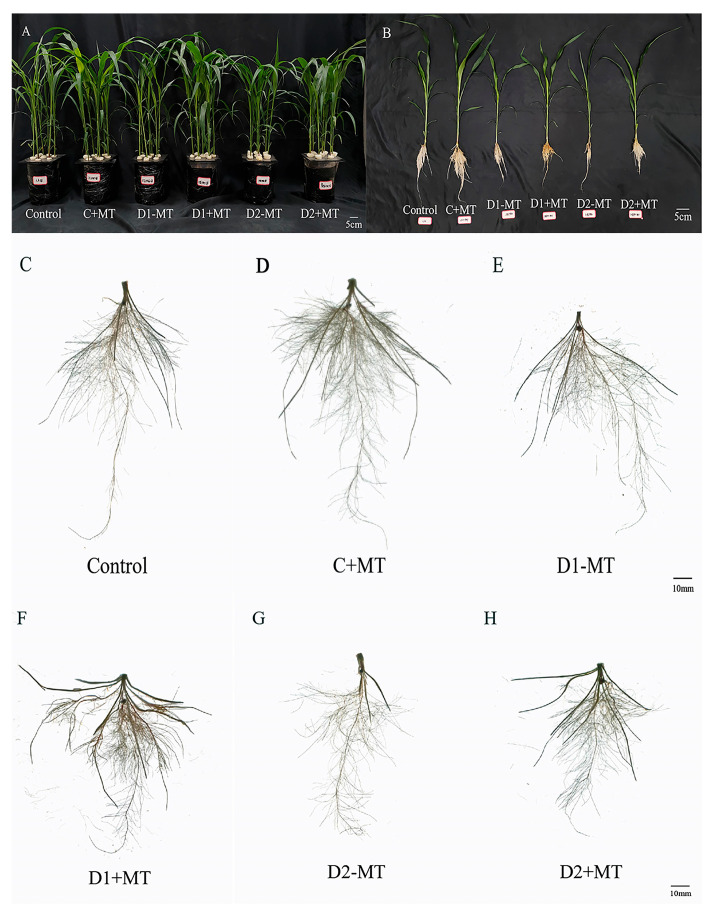
Growth Performance of Sweet Sorghum Seedlings Under Different Treatments. (**A**): Growth Performance of Sweet Sorghum Seedlings Under MT Treatment and Drought Stress; (**B**): Overall Growth Phenotype of Sweet Sorghum Seedlings Under MT Treatment and Drought Stress; (**C**): Scanning Images of sweet sorghum root system under normal conditions; (**D**): Scanning Images of sweet sorghum root system treated with 1 µmol·L^−1^ MT under normal conditions (C + MT); (**E**): Scanning Images of sweet sorghum root system under mild drought stress (D1-MT); (**F**): Scanning Images of sweet sorghum root system treated with 1 µmol·L^−1^ MT under mild drought stress (D1 + MT); (**G**): Scanning Images of sweet sorghum root system under moderate drought stress (D2-MT); (**H**): Scanning Images of sweet sorghum root system treated with 1 µmol·L^−1^ MT under moderate drought stress (D2 + MT).

Under varying treatment conditions, the growth status of sweet sorghum seedlings provides a clear indication of their morphological differences. As illustrated in Figure 1A, seedlings subjected to different drought stress treatments (D1-MT and D2-MT) exhibited a significantly reduced height compared to the control group. Notably, the application of 1 µmol·L^−1^ melatonin (MT) to the control group (C + MT) resulted in a marked increase in plant height. Additionally, seedlings exposed to different drought stress conditions demonstrated significantly enhanced growth following MT treatment. This suggests that 1 µmol·L^−1^ MT effectively mitigates drought-induced damage in plants. Further analysis reveals that MT also promotes root development in sweet sorghum (Figure 1B–H and Figure S1). Observations of scanned images of sweet sorghum roots under various treatment conditions indicate that the control group features well-developed root systems, whereas drought stress (D1-MT and D2-MT) leads to shorter roots and fewer lateral roots. In contrast, MT treatment (D1 + MT and D2 + MT) significantly counteracts the adverse effects of drought on root growth, as evidenced by improvements in root length, root tip quantity, and the number of lateral roots. Similar results were also observed in the phenotypic indicators and root indicators we measured (Figure S1), consistent with our results in the preliminary experiment (Figures S2–S4).

### 2.2. Exogenous MT Promotes Proline Synthesis in Leaves of Sweet Sorghum Seedlings Under Drought Stress

**Figure 2:**
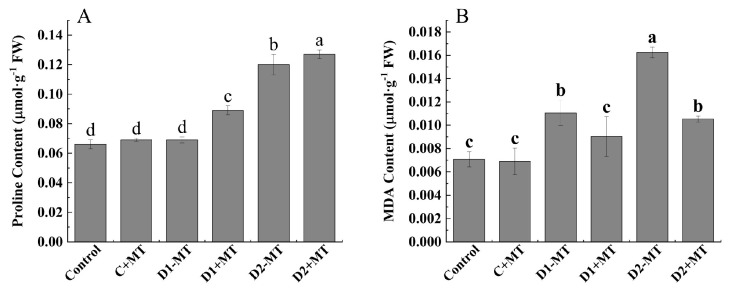
Changes in various drought response-related physiological parameters of sweet sorghum under MT treatment and drought stress. Note: C + MT: Control + 1 µmol·L^−1^ MT; D1-MT: 5% PEG-induced drought stress; D1 + MT: D1 + 1 µmol·L^−1^ MT; D2-MT: 10% PEG-induced drought stress; D2 + MT: D2 + 1 µmol·L^−1^ MT. (**A**): Effects of Exogenous MT on Proline Content under PEG-Induced Drought Conditions; (**B**): Effects of Exogenous MT on MDA Content Under PEG-Induced Drought. Data are presented as mean ± SD (*n* = 3). Different lowercase letters above the bars indicate significant differences among treatments at *p* < 0.05 according to one-way ANOVA followed by Duncan’s multiple range test.

In this study, we measured proline content in the leaves of sweet sorghum under varying drought conditions and following exogenous treatment with melatonin (MT). As illustrated in Figure 2A, drought stress significantly increased proline levels in sweet sorghum seedlings. Under hydroponic conditions, mild drought did not result in a notable increase in proline content; however, moderate drought conditions led to a significant increase of 80.73% in proline accumulation. Application of 1 µmol·L^−1^ MT under normal conditions resulted in a slight elevation in proline levels. In comparison to the drought stress conditions, treatment with 1 µmol·L^−1^ MT significantly enhanced proline content by 27.76% in seedlings subjected to mild drought and by 5.95% under moderate drought stress.

### 2.3. Exogenous MT Reduces Malondialdehyde Content in Leaves of Sweet Sorghum Seedlings Under Drought Stress

As shown in Figure 2B, drought stress significantly increased MDA content in sweet sorghum seedlings compared with the control group. Mild drought increased MDA content by 56.28%, and moderate drought increased it by 129.6%. Under normal conditions, application of 1 µmol·L^−1^ exogenous melatonin (MT) did not significantly affect MDA levels. Under drought stress, however, treatment with 1 µmol·L^−1^ MT significantly reduced MDA content by 18.33% under mild drought and by 35.18% under moderate drought, compared with the corresponding drought-only groups.

### 2.4. Exogenous MT Increases the Synthesis of Photosynthetic Pigments in Sweet Sorghum Under Drought Stress

**Figure 3:**
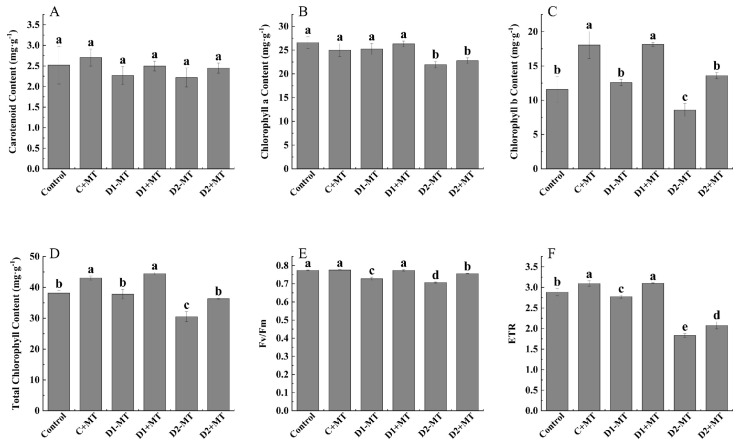
Photosynthetic response of sweet sorghum seedling leaves under MT treatment and drought stress. Note: Control; C + MT: Control + 1 µmol·L^−1^ MT; D1-MT: 5% PEG-induced drought stress; D1 + MT: D1 + 1 µmol·L^−1^ MT; D2-MT: 10% PEG-induced drought stress; D2 + MT: D2 + 1 µmol·L^−1^ MT. (**A**): Different drought levels and 1 µmol·L^−1^ MT under hydroponic treatment on carotenoid content in sweet sorghum leaves; (**B**): different drought levels and 1 µmol·L^−1^ MT under hydroponic treatment on chlorophyll a content in sweet sorghum leaves; (**C**): different drought levels and 1 µmol·L^−1^ MT under hydroponic treatment on chlorophyll b content in sweet sorghum leaves; (**D**): different drought levels and 1 µmol·L^−1^ MT under hydroponic treatment on total chlorophyll content in sweet sorghum leaves; (**E**): effects of different drought levels and 1 µmol·L^−1^ MT on Fv/Fm of sweet sorghum leaves under hydroponic treatment; (**F**): effects of different drought levels and 1 µmol·L^−1^ MT on ETR of sweet sorghum leaves under hydroponic treatment. The bar chart is plotted based on the mean values of three independent repeated experiments, and error bars represent standard deviation (SD). Different lowercase letters above the bars indicate significant differences at the 0.05 level (*p* < 0.05) according to one-way ANOVA followed by Duncan’s multiple range test.

Photosynthetic pigment contents in sweet sorghum leaves under different treatments are presented in Figure 3A–D. Under mild drought stress, no significant changes were observed in chlorophyll a, chlorophyll b, carotenoids, or total chlorophyll compared with the control. Under moderate drought stress, chlorophyll a, chlorophyll b, and total chlorophyll decreased significantly by 17.39%, 25.94%, and 16.91%, respectively. Under normal conditions, application of 1 µmol·L^−1^ melatonin (MT) did not significantly affect chlorophyll a or carotenoids, whereas chlorophyll b and total chlorophyll increased significantly by 55.75% and 18.82%, respectively. Under mild drought stress, MT treatment caused non-significant increases in chlorophyll a and carotenoids but significant increases in chlorophyll b (44.35%) and total chlorophyll (20.02%). Under moderate drought stress, MT treatment also resulted in non-significant changes in chlorophyll a and carotenoids, while chlorophyll b and total chlorophyll increased significantly by 58.42% and 17.77%, respectively.

### 2.5. Exogenous MT Enhances the Photosynthetic Efficiency of Chlorophyll in Sweet Sorghum Seedlings Under Drought

To investigate whether melatonin (MT) affects the photosynthetic efficiency of sweet sorghum under drought stress, this study further examined the impact of exogenous MT on chlorophyll fluorescence parameters in sweet sorghum seedlings subjected to PEG-simulated drought conditions. The variable Fv/Fm represents the maximum photochemical efficiency of the plants. As shown in Figure 3E, drought stress resulted in a decrease in Fv/Fm in sweet sorghum. Mild and moderate drought stress led to reductions in Fv/Fm of 5.97% and 8.64%, respectively. Under normal conditions, MT did not significantly alter Fv/Fm; however, in the presence of drought stress, the application of 1 µmol·L^−1^ MT significantly improved Fv/Fm by 6.31% and 6.92% under mild and moderate drought conditions, respectively. The electron transport rate (ETR) reflects the rate at which electrons are transferred during photosynthesis and directly influences the rate of photosynthesis. As illustrated in Figure 3F, under mild drought conditions, the ETR of sweet sorghum decreased by 3.85%, while it experienced a significant reduction of 36.45% under moderate drought stress. Under normal conditions, the application of 1 µmol·L^−1^ MT significantly enhanced the ETR of sweet sorghum leaves by 7.36%. Furthermore, under drought stress, the application of 1 µmol·L^−1^ MT resulted in significant increases in ETR of 11.85% and 13.23% for seedlings subjected to mild and moderate drought stress, respectively.

### 2.6. Exogenous Melatonin Modulates Antioxidant Enzyme Activity in Sweet Sorghum Under Drought Stress

**Figure 4:**
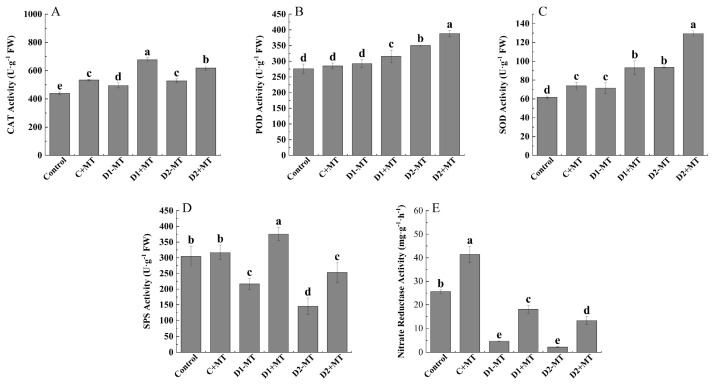
Changes in drought response-related enzyme activities in the root system of sweet sorghum under MT treatment and drought stress. Note: C + MT: Control + 1 µmol·L^−1^ MT; D1-MT: 5% PEG-induced drought stress; D1 + MT: D1 + 1 µmol·L^−1^ MT; D2-MT: 10% PEG-induced drought stress; D2 + MT: D2 + 1 µmol·L^−1^ MT. (**A**): Different drought levels and 1 µmol·L^−1^ MT under hydroponic treatment on catalase (CAT) activity in sweet sorghum roots; (**B**): different drought levels and 1 µmol·L^−1^ MT under hydroponic treatment on peroxidase (POD) activity in sweet sorghum roots; (**C**): different drought levels and 1 µmol·L^−1^ MT under hydroponic treatment on superoxide dismutase (SOD) activity in sweet sorghum roots; (**D**): different drought levels and 1 µmol·L^−1^ MT under hydroponic treatment on sucrose phosphate synthase (SPS) activity in sweet sorghum roots; (**E**): different drought levels and 1 µmol·L^−1^ MT under hydroponic treatment on nitrate reductase (NR) activity in sweet sorghum roots. The bar chart is plotted based on the mean values of three independent repeated experiments, and error bars represent standard deviation (SD). Different lowercase letters above the bars indicate significant differences at the 0.05 level (*p* < 0.05) according to one-way ANOVA followed by Duncan’s multiple range test.

Drought stress indirectly induces oxidative stress in the roots of sweet sorghum, prompting the plant to enhance the activity of key antioxidant enzymes in response. Under mild drought stress, the activities of catalase (CAT) and superoxide dismutase (SOD) in the roots of sweet sorghum significantly increased by 12.88% (Figure 4A) and 16.06% (Figure 4C), respectively, compared to the control group. Although the activity of peroxidase (POD) showed an upward trend, it did not reach statistical significance (Figure 4B). Under moderate drought stress, the activities of POD, SOD, and CAT in the roots increased significantly by 26.73%, 51.63%, and 20.15%, respectively, compared to the control. Under control conditions, exogenous application of 1 µmol·L^−1^ melatonin (MT) did not result in significant changes in POD activity; however, SOD and CAT activities were significantly elevated by 19.75% and 21.43%, respectively. Under mild drought stress, treatment with 1 µmol·L^−1^ MT significantly increased POD, SOD, and CAT activities by 8.02%, 30.12%, and 36.62%, respectively. Similarly, under moderate drought stress, MT treatment led to significant enhancements in POD, SOD, and CAT activities, with increases of 10.73%, 38.54%, and 17.33%, respectively. These results confirm that 1 µmol·L^−1^ MT effectively enhances the antioxidant capacity of sweet sorghum roots by differentially increasing the activities of POD, SOD, and CAT, thereby alleviating oxidative damage induced by drought stress.

### 2.7. Exogenous Melatonin Modulates Carbon and Nitrogen Metabolism-Related Enzyme Activities in Sweet Sorghum Under Drought Stress

As shown in Figure 4D, drought stress significantly inhibits sucrose synthesis in sweet sorghum seedlings. Under mild and moderate drought conditions, the activity of sucrose phosphate synthase (SPS) decreased by 28.90% and 52.38%, respectively, compared to the control group. Exogenous melatonin (MT) at 1 µmol·L^−1^ did not significantly affect SPS activity under normal moisture conditions. However, under mild drought stress, MT treatment significantly increased SPS activity by 73.37%, and this effect was further enhanced to 74.39% under moderate drought stress. Figure 4E illustrates that drought treatment markedly reduced nitrate reductase (NR) activity, with decreases of 81.67% and 91.53% under mild and moderate drought stress, respectively. Under normal moisture conditions, 1 µmol·L^−1^ MT treatment significantly increased NR activity by 61.35%. Notably, in comparison to drought stress alone, MT treatment significantly enhanced NR activity under both mild and moderate drought conditions, with increases of 285.48% and 512.74%, respectively. These results demonstrate that 1 µmol·L^−1^ MT effectively promotes sucrose synthesis and nitrogen uptake in sweet sorghum by significantly enhancing the activities of SPS and NR.

### 2.8. Transcriptome Sequencing Data Quality Assessment

In a transcriptome sequencing study focused on the root tissues of sweet sorghum after 7 days of treatment, three biological replicates were established for each condition: Control, D1-MT, D1 + MT, D2-MT, and D2 + MT, resulting in a total of 15 samples for eukaryotic reference transcriptome (RNA-seq) analysis. Following stringent quality control measures, we obtained a total of 93.65 Gb of high-quality clean data, with each sample achieving a minimum of 5.96 Gb of clean data. Furthermore, the Q30 base percentage for all samples was not less than 93.45%, indicating a high level of accuracy and reliability in the sequencing data. Clean reads from each sample were aligned with the designated reference genome, yielding high-quality results suitable for further analysis (Table S1).

### 2.9. Screening for Differential Expression Genes

**Figure 5:**
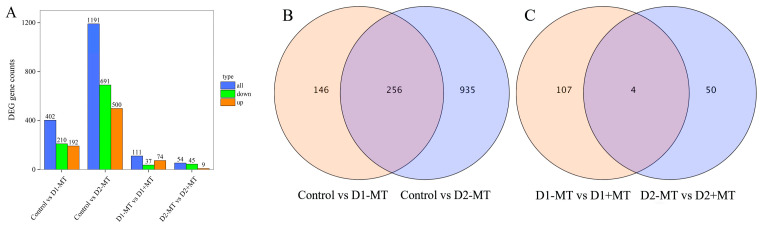
Bar chart of differentially expressed gene numbers and Venn diagram of differentially expressed genes. (**A**): Bar chart of the number of differentially expressed genes (DEGs) in four comparison groups: Control vs. D1-MT, Control vs. D2-MT, D1-MT vs. D1 + MT and D2-MT vs. D2 + MT. (**B**): Venn diagram of DEGs between two comparison groups: Control vs. D1-MT and Control vs. D2-MT. (**C**): Venn diagram of DEGs between two comparison groups: D1-MT vs. D1 + MT and D2-MT vs. D2 + MT.

Based on the transcriptome data, four comparisons—Control vs. D1-MT, Control vs. D2-MT, D1-MT vs. D1 + MT, and D2-MT vs. D2 + MT—were analyzed to identify differentially expressed genes (DEGs). The numbers of DEGs were 402 (192 up-regulated, 210 down-regulated) for Control vs. D1-MT, 1191 (500 up-regulated, 691 down-regulated) for Control vs. D2-MT, 111 (74 up-regulated, 37 down-regulated) for D1-MT vs. D1 + MT, and 54 (9 up-regulated, 45 down-regulated) for D2-MT vs. D2 + MT, with Control vs. D2-MT exhibiting the highest number of DEGs and D2-MT vs. D2 + MT the lowest (Figure 5A). For detailed information on up- and down-regulated genes, see Table S2. A total of 256 DEGs were responsive to drought stress under both D1-MT and D2-MT treatments (Figure 5B). Under D1 + MT and D2 + MT treatments, four DEGs were commonly involved in regulating sweet sorghum’s response to drought-induced damage (Figure 5C). These findings indicate that as drought intensity increases, sweet sorghum mitigates drought stress damage by modulating gene expression, involving diverse physiological and molecular pathways, including photosynthesis, hormone signaling, and antioxidant enzyme activity.

### 2.10. GO/KEGG Analysis of Differentially Expressed Genes and Transcription Factors Mediating Drought Tolerance in Sorghum

Gene Ontology (GO) analysis was performed by annotating genes using the GO annotation system, followed by classification of differentially expressed genes (DEGs) into Biological Process (BP), Cellular Component (CC), and Molecular Function (MF) categories. The DEGs with similar functions were clustered, and GO enrichment bar plots provided a clear visualization of DEG distribution across these categories. This study primarily focused on the DEGs between the D1-MT vs. D1 + MT and D2-MT vs. D2 + MT comparisons. For D1-MT vs. D1 + MT, DEGs in the BP category were significantly enriched (*p* < 0.05) in processes related to primary and secondary cell wall biogenesis and cellulose biosynthesis (Figure S5A). In the CC category, enrichment was observed in plasma membrane components and membrane-associated processes (Figure S5B). In terms of MF, DEGs were significantly enriched in cellulose synthase activity and ADP binding (Figure S5C). For D2-MT vs. D2 + MT, DEGs in the BP category were predominantly enriched in ammonium ion transmembrane transport, hydrogen peroxide catabolism, and oxidative stress responses (Figure S5D). In the CC category, DEGs were enriched in plasma membrane components (Figure S5E), while in the MF category, ATPase activity and ATP binding were notably enriched (Figure S5F).

To investigate the key metabolic pathways of MT involved in drought stress in sweet sorghum, KEGG pathway enrichment analysis was performed on differentially expressed genes (DEGs). The enrichment analysis for the D1-MT vs. D1 + MT and D2-MT vs. D2 + MT comparisons revealed that DEGs were predominantly associated with glycolysis/gluconeogenesis, carbon fixation, general carbon metabolism, and plant hormone signal transduction pathways in photosynthetic organisms (Figure S6A). Notably, in the D2-MT vs. D2 + MT comparison, DEGs were significantly enriched in phenylpropanoid biosynthesis and amino acid biosynthesis pathways. In addition, pathways related to nitrogen metabolism, pentose and glucuronate interconversions, glycolysis/gluconeogenesis, and carbon metabolism were also highlighted as critical (Figure S6B).

Transcription factors (TFs) are proteins that bind to specific DNA sequences to regulate the transcription of target genes, playing a pivotal role in plant responses to environmental stimuli. These factors are commonly categorized into distinct families based on their functional domains. In this study, a total of seven TFs were identified in the comparison of D1-MT vs. D1 + MT, representing the AGC_RSK-2, B3-ARF, CAMK_CAMKL-CHK1, MYB, RLK-Pelle_WAK, TAZ, and TKL-PI-4 families. These TFs were significantly involved in modulating MT-mediated responses to mild drought stress (Figure S6C). In the comparison of D2-MT vs. D2 + MT, nine TFs were detected, belonging to the C2C2-Dof, CMGC_SRPK, HB-HD-ZIP, LOB, OFP, RLK-Pelle_CR4L, RLK-Pelle_LRR-VI-2, RLK-Pelle_LRR-XII-1, and bHLH families. These TFs were prominently implicated in the regulation of MT’s response to moderate drought stress (Figure S6D). The results suggest that, in response to drought stress, differential genes in sweet sorghum roots are primarily enriched in phenylpropanoid biosynthesis, amino acid metabolism, and starch–sucrose metabolism pathways. This indicates that sweet sorghum utilizes these pathways to mitigate the effects of drought stress. After the application of 1 μmol·L^−1^ MT, differential genes were predominantly enriched in pathways involved in amino acid synthesis, photosynthesis, carbon and nitrogen metabolism, and hormone signal transduction. These findings imply that MT alleviates drought-stress-induced damage in sweet sorghum by regulating these critical metabolic and signaling pathways.

### 2.11. Melatonin Enhances Drought Tolerance in Sweet Sorghum by Regulating Photosynthesis, Osmotic Homeostasis, Nitrogen Assimilation, Antioxidant Defense, and Hormone Signaling Through Transcriptome Reprogramming

To further elucidate the molecular mechanisms by which melatonin alleviates drought stress in sweet sorghum, this study systematically investigated the effects of melatonin on multiple key metabolic and signaling pathways in roots based on KEGG annotation and enrichment analysis of differentially expressed genes (DEGs) (detailed gene information is provided in Supplementary Table S3–S12).

In photosynthesis-related pathways, DEGs in the D1-MT vs. D1 + MT comparison (mild drought stress) were predominantly enriched in catalase isoenzymes. Their upregulation likely mitigates drought-induced oxidative damage, thereby maintaining normal carbon fixation and assimilation processes. In contrast, in the D2-MT vs. D2 + MT comparison (moderate drought stress), DEGs were mainly involved in root carbon fixation and metabolism pathways. Their downregulation suggests that melatonin may reduce photosynthesis-related energy expenditure in roots, reallocating resources to other adaptive physiological processes. Regarding osmotic adjustment, DEGs associated with arginine and proline metabolism, starch and sucrose metabolism, glycolysis, and gluconeogenesis were significantly enriched in the D1-MT vs. D1 + MT group, with eight genes upregulated, indicating that melatonin promotes the accumulation of osmolytes such as soluble sugars and proline. In the D2-MT vs. D2 + MT group, genes in these pathways were downregulated, suggesting a shift in energy metabolism strategies under more severe drought stress. For nitrogen metabolism, glutamate dehydrogenase and nitrate reductase genes were upregulated in D1-MT vs. D1 + MT, enhancing nitrogen uptake and assimilation in roots. In D2-MT vs. D2 + MT, however, genes encoding nitrate transporters were downregulated, indicating that melatonin dynamically modulates nitrogen homeostasis to alleviate stress-induced damage. In antioxidant-related pathways, DEGs were primarily enriched in non-enzymatic pathways, including ascorbate and aldarate metabolism, inositol phosphate metabolism, and phenylpropanoid and flavonoid biosynthesis. Among the enzymatic antioxidant systems, peroxidases were predominant, with more genes downregulated than upregulated, suggesting that melatonin coordinates both enzymatic and non-enzymatic systems to mitigate drought-induced oxidative stress. Additionally, in hormone signaling pathways, five genes, including auxin response factors and serine/threonine protein kinases, were upregulated in D1-MT vs. D1 + MT, mainly contributing to IAA and GA_3_ biosynthesis. In D2-MT vs. D2 + MT, genes associated with bHLH19, a transcription factor regulating GA_3_ biosynthesis, were downregulated, indicating that melatonin dynamically modulates endogenous hormone balance to adapt to varying intensities of drought stress. Collectively, melatonin orchestrates global transcriptional reprogramming, synergistically regulating photosynthesis, osmotic adjustment, nitrogen metabolism, antioxidant defense, and hormone signaling networks, thereby systematically enhancing drought tolerance in sweet sorghum.

## 3. Discussion

This study combines physiological and transcriptomic analyses to systematically elucidate the multifaceted mechanisms by which exogenous melatonin (MT) mitigates PEG-induced drought stress in sorghum seedlings. MT treatment markedly improved plant growth under drought conditions (Figure 1 and Figures S1–S4), enhanced the accumulation of photosynthetic pigments and photochemical efficiency (Figure 3), boosted antioxidant enzyme activities and osmotic adjustment capacity, and alleviated membrane lipid peroxidation (Figure 4). At the same time, findings suggest that 1 µmol·L^−1^ MT enhances proline accumulation in the leaves of sweet sorghum, aiding in the maintenance of osmotic balance to mitigate the effects of drought stress (Figure 2A) At the same time, a correlation analysis of growth indicators showed that in sweet sorghum seedlings under drought stress, a more “compact” root system (higher dry weight, lower volume) benefits aboveground growth (Figure S7). Malondialdehyde (MDA) is a product of membrane lipid peroxidation resulting from stress in plants, with higher MDA levels indicating greater damage to plant cell membranes this study [20]. In the present study exogenous melatonin significantly reduced MDA accumulation under drought stress (Figure 2B), suggesting that MT alleviates oxidative membrane damage.

Transcriptome profiling further revealed that MT orchestrates a global reprogramming of transcriptional networks, coordinately modulating pathways associated with photosynthesis, carbon and nitrogen metabolism, antioxidant defense, and hormone signaling. Collectively, these findings demonstrate that MT confers drought resilience in sorghum through integrated physiological and molecular regulation.

Drought-induced inhibition of photosynthesis is one of the primary factors limiting plant growth [20,21,22]. In this study, melatonin (MT) treatment significantly enhanced chlorophyll b content and chlorophyll fluorescence parameters (Fv/Fm) in sorghum seedlings, indicating that MT effectively protects the photosynthetic apparatus from drought-induced photooxidative damage. This finding is consistent with the results of Khan (2024) in wheat [22], which demonstrated that exogenous MT alleviates drought-induced suppression of photosynthetic carbon assimilation by upregulating the expression of photosynthesis-related genes and enhancing RuBP Case activity [23,24]. Notably, we observed a significant upregulation of several differentially expressed genes associated with photosystem II reaction center proteins and the electron transport chain following MT treatment, suggesting that MT may directly regulate the assembly and repair of the photosynthetic machinery at the transcriptional level. Additionally, the increased synthesis of photosynthetic pigments further ensured efficient light capture and energy conversion, providing sufficient energy for carbon assimilation [25,26].

Osmotic adjustment is a key strategy by which plants cope with water deficit, and proline, as a major compatible solute, contributes to maintaining cell turgor and membrane stability [27]. In the present study, MT treatment markedly enhanced proline accumulation and regulated key genes in the arginine and proline metabolism pathway, including P5CS (delta-1-pyrroline-5-carboxylate synthase) (Table S2B), consistent with observations in wheat [28,29] and maize [30]. Concurrently, MT application reduced MDA content, indicating alleviation of membrane lipid peroxidation, likely due to activation of the ROS-scavenging system. Activities of antioxidant enzymes, including SOD, POD, and CAT, were significantly elevated, and genes involved in the ascorbate–glutathione cycle were induced, collectively forming an MT-mediated protective network against oxidative damage [31,32,33]. Interestingly, non-enzymatic antioxidant systems, such as flavonoid and phenylpropanoid biosynthesis pathways, were also significantly enriched following MT treatment, suggesting that MT confers oxidative stress tolerance through the coordinated action of both enzymatic and non-enzymatic antioxidant mechanisms [34].

Carbon and nitrogen metabolism balance is crucial for plant adaptability under stress [35,36]. In the present study, MT treatment influenced the expression of genes related to starch and sucrose metabolism, promoting the accumulation of soluble sugars, which not only provides a substrate for osmotic regulation but also serves as a source for energy metabolism. This finding is consistent with the results of Cao et al. (2024) [15] in maize, which demonstrated that MT enhances drought tolerance by modulating the activity of carbon metabolism enzymes. Regarding nitrogen metabolism, MT upregulated the expression of glutamate dehydrogenase (GDH) and nitrate reductase (NR), thereby promoting nitrogen absorption and assimilation. Under severe stress, however, the downregulation of nitrate transporter genes suggests that MT may maintain root nitrogen balance by reducing the translocation of nitrogen to the shoot, thus prioritizing root function [37,38].

Transcriptome analysis provided critical insights into the molecular regulatory network of MT. GO and KEGG enrichment analyses (Figures S5 and S6, Tables S3–S12) revealed that differentially expressed genes (DEGs) were significantly enriched in pathways related to plant hormone signal transduction, MAPK signaling, phenylpropanoid biosynthesis, and flavonoid biosynthesis. The upregulation of genes associated with auxin (IAA) and gibberellin (GA_3_) signaling pathways corresponded with the observed promotion of root development and biomass accumulation, supporting the role of MT in enhancing root growth and water uptake efficiency [7,18]. Furthermore, differential expression of transcription factor families, including bHLH, MYB, NAC, and WRKY, indicated that MT orchestrates downstream transcriptional cascades to integrate environmental cues with growth and developmental programs [39,40,41,42]. Notably, several core transcription factors implicated in the coordinated regulation of photosynthesis, antioxidant defense, and hormone signaling, such as bHLH19 and MYB-related genes, were identified, suggesting that these factors may serve as pivotal nodes in the MT-mediated drought tolerance network.

Transcriptome sequencing was not performed for the MT (melatonin alone without stress, C + MT) treatment. Thus, the genome-wide transcriptional effects of melatonin under non-stressed conditions remain unknown. Nevertheless, our physiological data showed no significant changes in growth, Proline Content, MDA Content, or peroxidase activity in C + MT plants compared with controls (Figure 2 and Figure 4B), suggesting that melatonin exerts a more prominent regulatory role under drought stress. Future studies should include C + MT transcriptomic analysis to fully decipher the pleiotropic functions of melatonin in sweet sorghum.

## 4. Materials and Methods

### 4.1. Materials

The experimental material used in this study was the sweet sorghum cultivar “Dali Shi,” which was obtained from Bai green International Grass Industry Co., Ltd. (Beijing, China). Melatonin (MT) was sourced from Beijing Solebao Technology Co., Ltd., (Beijing, China) while polyethylene glycol 6000 (PEG6000) was procured from Shandong Keyuan Biochemical Co., Ltd. (Shandong, China) The nutrient solution was prepared according to the Hoagland formula, acquired from Henan Caiju Dongli Agricultural Technology Co., Ltd. (Henan, China). Furthermore, catalase (CAT) and superoxide dismutase (SOD) assay kits (the item numbers are BC0200, BC0170) were purchased from Beijing Solebao Technology Co., Ltd. (Beijing, China). Sulfosalicylic acid, acidic ninhydrin, toluene, phosphate buffer, guaiacol, and potassium dihydrogen phosphate were purchased from Henan Cai ju Dong li Agricultural Technology Co., Ltd. (Henan, China).

### 4.2. Preliminary Screening for Melatonin Concentration

Mild drought stress (D1-MT) was simulated with 5% PEG, and moderate drought stress (D2-MT) with 10% PEG. Melatonin (MT) was applied at two concentrations, 1 μmol·L^−1^ (MT1) and 10 μmol·L^−1^ (MT10), resulting in four treatment combinations: D1 + MT1, D1 + MT10, D2 + MT1, and D2 + MT10. The control group was treated with nutrient solution only.

Plump and uniformly sized seeds were selected, sterilized with 5% sodium hypochlorite solution for 20 min, rinsed thoroughly with distilled water, and air-dried naturally. The seeds were then imbibed in distilled water in the dark for 4 h. After imbibition, seeds were placed evenly on PVC mesh plates positioned over plastic boxes filled with distilled water, allowing contact with water, and germination was conducted in the dark. After germination, light treatment was initiated under a photoperiod of 12 h light/12 h dark, light intensity of 6600 lux, and day/night temperature of 27 °C/25 °C. During germination, the nutrient solution was replaced every 3 days as follows: first with distilled water, second with quarter-strength nutrient solution, and third with half-strength nutrient solution. When the shoot length reached approximately 2–3 cm, uniformly growing seedlings were selected and transferred to containers with 1 L of half-strength nutrient solution, which was replaced every 3 days for two changes, followed by full-strength nutrient solution thereafter.

### 4.3. Treatment Methods

To simulate mild and moderate drought conditions, 5% and 10% polyethylene glycol 6000 (PEG) were used, respectively. Preliminary experiments were designed with melatonin (MT) concentrations of 1 µmol·L^−1^ and 10 µmol·L^−1^, and based on the results (Figures S2–S4), 1 µmol·L^−1^ MT was selected. Initially, seeds were soaked in distilled water for 4 h to induce germination, followed by light treatment. The nutrient solution was changed every 3 days, using distilled water for the first change, a quarter-strength nutrient solution for the second change, and a half-strength nutrient solution for the third change. When the germination length reached 2–3 cm, seedlings with consistent growth were selected and transferred to containers with 1 L of half-strength nutrient solution for continued cultivation. Subsequently, two more changes of nutrient solution were performed, ultimately using full-strength nutrient solution. Treatment commenced when the seedlings developed four leaves and one heart. Using the root application method, melatonin was added to the Hogland solution corresponding to each treatment group, with the following groups set up: Control (1 L Hogland solution); C + MT (Control with 1 µmol·L^−1^ MT added); D1-MT (5% PEG to simulate drought); D1 + MT (1 µmol·L^−1^ MT added to D1-MT treatment); D2-MT (10% PEG to simulate drought); D2 + MT (1 µmol·L^−1^ MT added to D2-MT treatment). After 7 days of treatment, the fourth true leaf from the bottom upwards and root tissues were collected. Various physiological responses of the plants to PEG-simulated drought conditions were assessed by measuring the osmotic adjustment capacity of the leaf tissue, membrane injury levels, photosynthetic system functionality, root morphology and vitality, activities of enzymes related to carbon and nitrogen metabolism, and antioxidant enzyme activities, and by conducting transcriptome sequencing.

### 4.4. Determination of Proline Content

Proline content was determined according to the acidic ninhydrin method described by Bates et al. (1973) [43]. Leaf samples (1 g) were homogenized with 10 mL of 30 g·L^−1^ sulfosalicylic acid. The homogenate was heated in boiling water for 10 min, cooled, and centrifuged at 3000 r·min^−1^. An aliquot of the supernatant (4 mL) was mixed successively with 4 mL of distilled water, 4 mL of glacial acetic acid, and 8 mL of 25 g·L^−1^ acidic ninhydrin reagent. The mixture was incubated in boiling water for 1 h and then cooled. Subsequently, 8 mL of toluene was added, and the two phases were allowed to separate. The red toluene phase (containing the proline–ninhydrin complex) was collected for absorbance measurement at 520 nm. Proline concentration was calculated using a standard curve (Figure S8). Proline content (μmol·g^−1^ FW) = (ρ × V2)/(V1 × m). The absorbance was measured at 520 nm. ρ is the proline content obtained from the standard curve; V2 is the total volume of the extract (mL); V1 is the volume of the solution to be measured (mL); and m is the sample mass.

### 4.5. Determination of Malondialdehyde Content

Malondialdehyde (MDA) content was determined using thiobarbituric acid (TBA) as described by Heath and Packer [44]. Fresh leaf samples (1 g) were homogenized with 10 mL of 10% (*w*/*v*) trichloroacetic acid (TCA). The homogenate was centrifuged at 4000 r·min^−1^, and 4 mL of the supernatant was mixed with 4 mL of 0.6% (*w*/*v*) thiobarbituric acid (prepared in 10% TCA). The mixture was heated in boiling water for 15 min, rapidly cooled, and then centrifuged. The absorbance of the supernatant was measured at 450 nm and 532 nm. The MDA concentration was calculated using the following formula: MDA content (μmol/g FW) = (c × V)/(m × 1000); c = 6.45(OD_532_ − OD_600_) − 0.56 OD_450,_ where c is the MDA concentration (μmol/L); V is the volume of the extract (5 mL); and m is the fresh weight of plant tissue (g).

### 4.6. Determination of Photosynthetic Pigment Content

Chlorophyll and carotenoid contents were determined using the ethanol extraction method by Hartmut [45]. The fourth true leaf from the bottom of sweet sorghum seedlings was collected. A 0.1 g leaf sample was cut into small pieces and completely immersed in 95% (*v*/*v*) ethanol, with the final volume adjusted to 10 mL. The sample was kept in the dark at room temperature for 48 h until the leaf fragments were completely decolorized and turned white. Absorbance was measured at 665 nm, 649 nm, and 470 nm using 95% ethanol as a blank. Each measurement was performed in triplicate. The chlorophyll a (C_a_), chlorophyll b (C_b_), and total carotenoid (Car) concentrations were calculated using the following equations:

C_a_ (mg/g FW) = 13.95 × A_665_ − 6.88 × A_649_

C_b_ (mg/g FW) = 24.96 × A_649_ − 7.32 × A_665_

Car (mg/g FW) = (1000 × A_470_ − 2.05 × C_a_ − 114.8 × C_b_)/245

Total chlorophyll (mg/g FW) = C_a_ + C_b_

### 4.7. Chlorophyll Fluorescence Parameter Measurement

Before measurement, the fourth true leaf of sweet sorghum seedlings from the bottom up was dark-adapted for 20 min, and chlorophyll fluorescence parameters were measured using a chlorophyll fluorometer (FMS-2, Hansa Scientific Instruments Co., Ltd. Norfolk, UK). Basic data such as Fo, Fm and Fv were obtained. Then, indicators such as the maximum photochemical efficiency of PSII reaction centers (Fv/Fm) and electron transport rate (ETR) were calculated.

### 4.8. Nitrate Reductase Activity Assay

Nitrate reductase activity was determined using the sulfanilamide colorimetric method as described by Jaworski [46]. Two 0.5 g samples of root tissue were prepared; one sample was mixed with 5 mL of 0.1 mol·L^−1^ phosphate buffer and 5 mL of 0.2 mol·L^−1^ KNO_3_ solution, while the other sample had the KNO_3_ solution replaced with distilled water to serve as a control. The samples were then subjected to vacuum using a vacuum pump to allow the material to settle. Following this, the samples were incubated in a water bath at 30 °C for 30 min. For the experimental group, 1 mL of 30% trichloroacetic acid solution was added, while the control group received the trichloroacetic acid before vacuum treatment. Afterward, 1 mL of each sample was taken, and 2 mL of sulfanilamide reagent and 2 mL of α-naphthylamine reagent were added, followed by thorough mixing and a 30 min standing period. The absorbance was measured at 520 nm, and nitrate reductase activity was calculated using a standard curve (Figure S9). The calculation formula is as follows: Nitrate reductase activity (μg·g^−1^·h^−1^) = [ρ/(m × t)] × 10. ρ is the concentration of NO^−2^ (μg/mL); m is the fresh weight of the material; t is the reaction time in minutes; 10 is the initial solution volume.

### 4.9. Measurement of Catalase Activity, Superoxide Dismutase Activity, Sucrose Phosphate Synthase Activity and Peroxidase Activity

Catalase activity, superoxide dismutase activity, and sucrose phosphate synthase activity were measured according to the instructions provided in the assay kits (the item numbers are BC0095, BC5165, BC0600). The measurements were carried out using kits purchased from Beijing Solarbio Technology Co., Ltd. (Beijing, China). Concentrations were calculated using the following equations: CAT(U/g) = [ΔA × V_total reaction_/(ε × d) × 10^6^] ÷ (W × V_sample_/V_total sample_) ÷ T = 678 × ΔA ÷ W. V_total reaction_: total volume of the reaction system, 1.035 × 10^−3^ L; ε: molar absorption coefficient of H_2_O_2_, 43.6 L/mol/cm; d: path length of 1 mL quartz cuvette, 1 cm; V sample: volume of sample added, 0.035 mL; V_total sample_: volume of extraction solution added, 1 mL; T: reaction time, 1 min; W: sample weight, g; ΔA = A1 − A2.

Peroxidase (POD) activity was determined using the guaiacol method as described by Chance and Maehly [47]. Fresh leaf samples (1 g) were homogenized with 10 mL of 20 mmol·L^−1^ KH_2_PO_4_ solution. The homogenate was centrifuged at 4000 r·min^−1^, and 2 mL of the supernatant was mixed with 6 mL of reaction mixture containing 100 mmol·L^−1^ phosphate buffer (pH 7.0), guaiacol, and 30% H_2_O_2_. A blank was prepared using KH_2_PO_4_ solution instead of the supernatant. The absorbance was immediately measured at 470 nm and recorded every minute for 3–5 min. POD activity was calculated based on the increase in absorbance per minute, using the extinction coefficient of tetraguaiacol (ε = 26.6 mmol·L^−1^·cm^−1^) as follows: POD activity (U⋅g^−1^ FW) = (Δ*A*_470_ × *V*_extract_)/Δ*t* × *W* × *a*. among, ΔA470 = change in absorbance per minute (min^−1^); V extract = volume of enzyme extract added (mL); *W* = sample fresh weight (g); a = aliquot of enzyme extract used (mL).

### 4.10. RNA Extraction

Total RNA was extracted from root samples using Trizol reagent (Invitrogen, Carlsbad, CA, USA) according to the manufacturer’s protocol. Root tissues from three independent biological replicates of each treatment group (control, D1-MT, D2-MT, D1 + MT, D2 + MT), each containing three technical replicates, were collected, immediately frozen in liquid nitrogen, and stored at −80 °C until processing. The RNA extraction procedure was based on the acid guanidinium thiocyanate–phenol–chloroform method [48], and the quality and integrity of the extracted RNA were assessed prior to sequencing.

### 4.11. Transcriptome Sequencing and Differential Gene Analysis

Transcriptome sequencing was conducted by Beijing Biomarker Biotech Co., Ltd. (Beijing, China). using the Illumina HiSeq 2500 platform. Differential gene expression analysis was performed based on the count values obtained for each sample. For sample groups with biological replicates, we utilized DESeq2 software (version R 4.0.2) for differential analysis. Differentially expressed genes were identified using the criteria of Fold Change (FC) ≥ 2 and a false discovery rate (FDR) < 0.01. In this context, Fold Change refers to the ratio of expression levels between two groups of samples, with its logarithm (log2FC) used for comparison to reflect the magnitude of changes in gene expression. The FDR represents the adjusted significance level of the differential significance *p*-value. Specifically, a greater absolute value of log2FC and a smaller FDR indicate a more significant biological relevance of the differential expression observed for the gene. Statistical analyses were performed using SPSS version 26.0, and graphical representations were created using GraphPad Prism version 9.0. Data are presented as mean ± standard deviation (SD), with a significance level set at *p* < 0.05.

### 4.12. Data Analysis

Data were organized using Excel 2019 and analyzed with IBM SPSS Statistics 27.0 software, with Duncan’s method used for multiple comparisons (*p* < 0.05). Charts were produced using Origin 2022 software.

## 5. Conclusions

This study constructed a multi-layered regulatory model for exogenous MT-mediated drought tolerance in sweet sorghum. At the physiological level, MT effectively alleviated drought-induced oxidative stress by promoting the accumulation of osmotic regulators (proline) and reducing plasma membrane damage (MDA), thereby inhibiting lipid peroxidation and maintaining cellular homeostasis. Additionally, MT enhanced leaf photosynthetic pigment content, increased maximum photochemical efficiency (Fv/Fm), and increased the electron transport rate (ETR), thereby mitigating damage to Photosystem II. The activities of key carbon and nitrogen metabolism enzymes (SPS, NR) and antioxidant enzymes (POD, SOD, CAT) were also significantly upregulated. At the transcriptomic level, differentially expressed genes (DEGs) that responded to drought stress were primarily enriched in pathways associated with stress responses, including carbon fixation, glycolysis/gluconeogenesis, nitrogen metabolism, antioxidant defense, and plant hormone signal transduction. MT upregulated the expression of genes such as peroxidase isoenzyme (SORBI_3004G011566), proline dehydrogenase (SORBI_3001G304700), and glutamate dehydrogenase (SORBI_3006G165400), promoting the accumulation of osmotic regulators and improving nitrogen metabolism efficiency. MT also activated antioxidant defense-related genes (peroxidase SORBI_3006G277500), reducing reactive oxygen species (ROS) accumulation. Furthermore, MT modulated the expression of transcription factor families, including bHLH, MYB, and RLK-Pelle, coordinating auxin and gibberellin signaling and forming a multi-level molecular regulatory network for drought stress response. Collectively, MT maintains the physiological integrity of sweet sorghum seedlings by enhancing osmotic regulation, photosynthetic protection, and physiological activities (e.g., SPS, NR, POD, SOD, and CAT) while regulating gene expression and metabolic pathways to modulate drought stress responses. This study provides a theoretical basis for the application of MT in drought-prone forage crops and offers insights into the functional roles of key genes involved in MT-mediated drought stress response in sweet sorghum.

## Data Availability

The original contributions presented in this study are included in the article/Supplementary Material. The transcriptome data generated in this study have been deposited in the NCBI database under the BioProject accession number PRJNA1471139.

## Supplementary Materials

The following supporting information can be downloaded at https://www.mdpi.com/article/10.3390/ijms27125291/s1.
