# Supplementary material for: Melatonin Alleviates Drought Stress in Sweet Sorghum Seedlings via Protection of Photosynthetic Apparatus and Carbon-Nitrogen Metabolism
Source: Int J Mol Sci. 2026 Jun 11;27(12):5291. doi: 10.3390/ijms27125291 (PMC13299841; doi:10.3390/ijms27125291)
Supplement: Supplementary file 1 [file ijms-27-05291-s001.zip › IJMS-supplement figure vs table.pdf]

Supplement

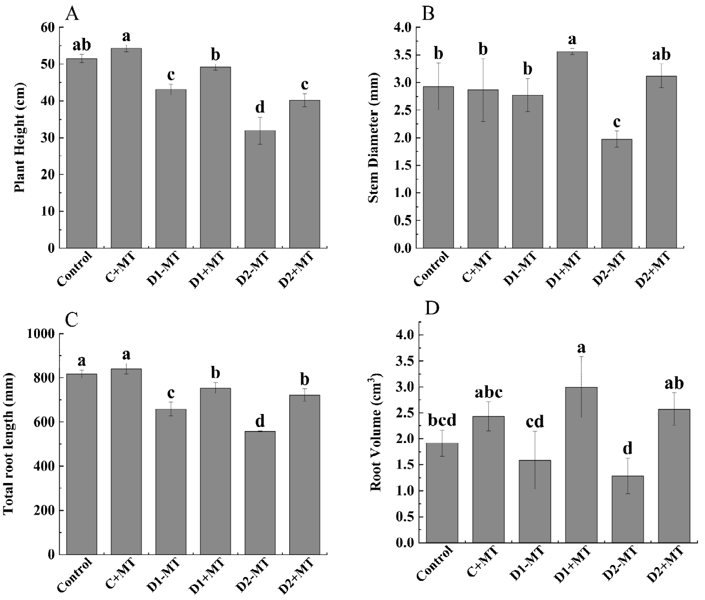

Figure S1. Effects of melatonin on sweet sorghum growth and roots under drought stress. Control: Normal watering; C+MT: 1 μmol·L^{-1} melatonin treatment under control conditions; D1-MT: Mild drought stress (without melatonin); D1+MT1: Mild drought stress with 1 μmol·L^{-1} melatonin treatment; D2-MT: Moderate drought stress (without melatonin); D2+MT: Moderate drought stress with 1 μmol·L^{-1} melatonin treatment. A: Effects of different drought stresses and 1 μmol·L^{-1} MT treatment on the plant height of sweet sorghum; B: Effects of different drought stresses and 1 μmol·L^{-1} MT treatment on the stem thickness of sweet sorghum; C: Effects of different drought stresses and 1 μmol·L^{-1} MT treatment on the total root length of sweet sorghum; D: Effects of different drought stresses and 1 μmol·L^{-1} MT treatment on the root volume of sweet sorghum. Significant differences are indicated by different lowercase letters（$P < 0.05$）.

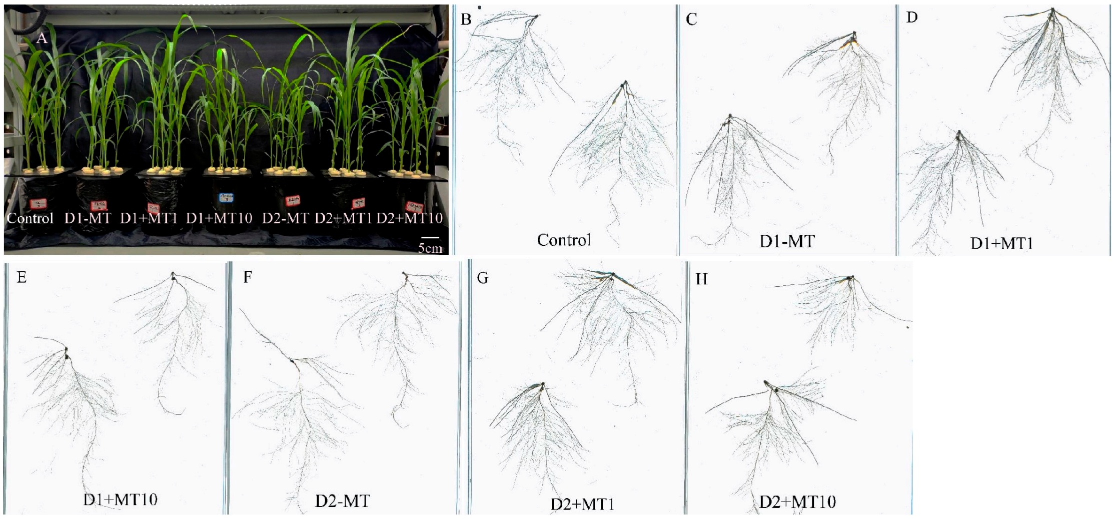

Figure S2. Growth phenotypes of sweet sorghum seedlings under different treatments.

Control: well-watered Hoagland control; D1-MT: mild drought stress without melatonin; D1+MT1: mild drought stress + 1 μmol·L^{-1} melatonin; D1+MT10: mild drought stress + 10 μmol·L^{-1} melatonin; D2-MT: moderate drought stress without melatonin; D2+MT1: moderate drought stress + 1 μmol·L^{-1} melatonin; D2+MT10: moderate drought stress + 10 μmol·L^{-1} melatonin. Photographs were taken after 7 days of treatment. Scale bar = 5 cm. A: Growth phenotypes of sweet sorghum under different treatments. B: Root growth phenotypes of sweet sorghum under different treatments.

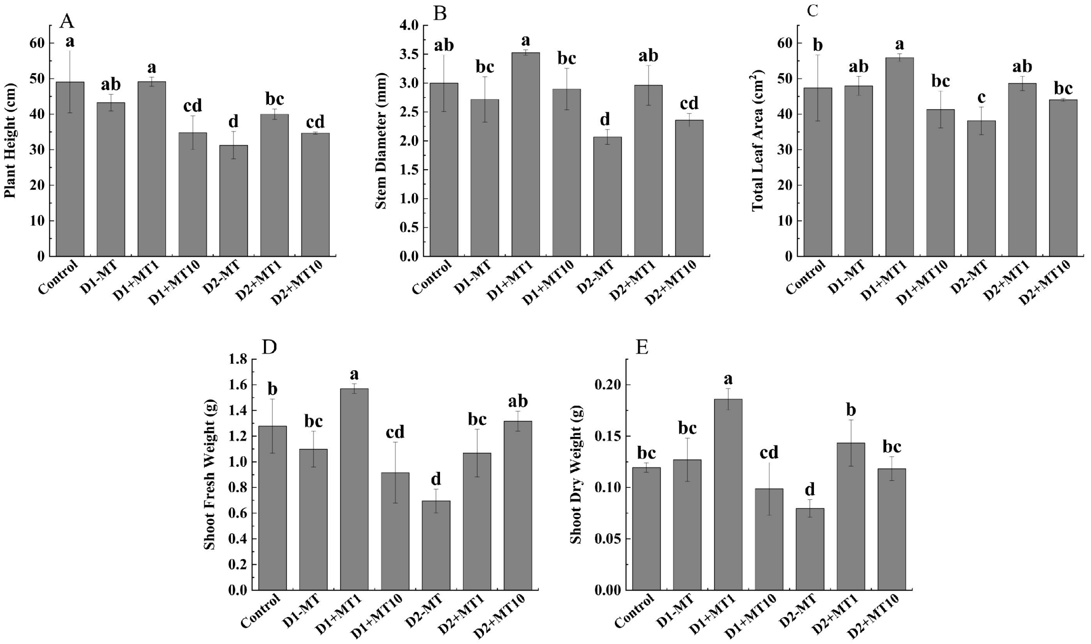

Figure S3. Effects of different treatments on sweet sorghum growth phenotypic traits.

Control: well-watered Hoagland control; D1-MT: mild drought stress without melatonin; D1+MT1: mild drought stress + 1 μmol·L^{-1} melatonin; D1+MT10: mild drought stress + 10 μmol·L^{-1} melatonin; D2-MT: moderate drought stress without melatonin; D2+MT1: moderate drought stress + 1 μmol·L^{-1} melatonin; D2+MT10: moderate drought stress + 10 μmol·L^{-1} melatonin. A: plant height; B: Stem Diameter; C: Total Leaf Area; D: Shoot Fresh Weight; E: Shoot Dry Weight.

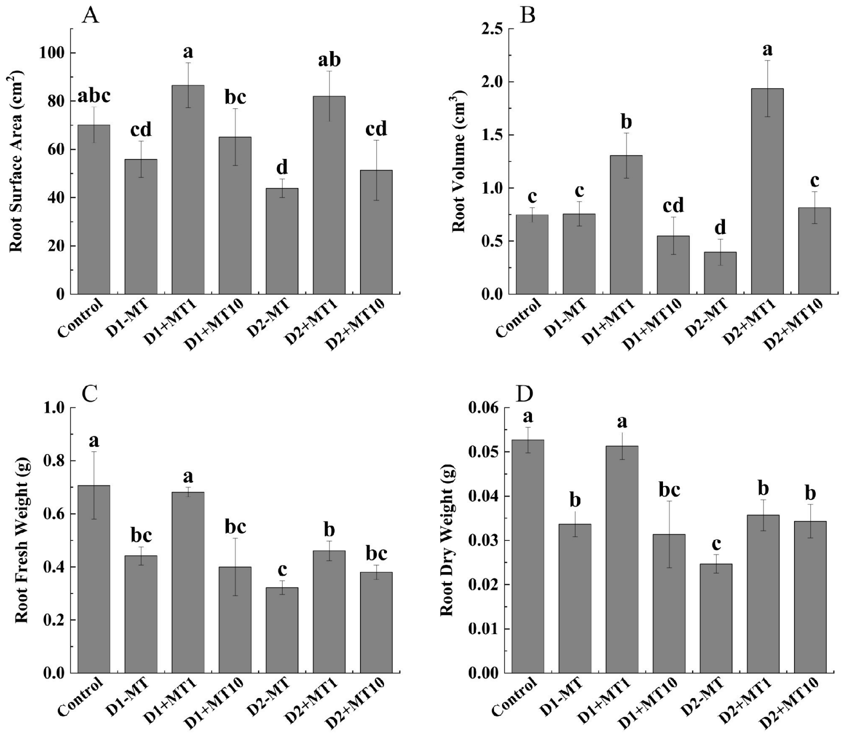

Figure S4. The effects of different treatments on sweet sorghum root growth

Control: well-watered Hoagland control; D1-MT: mild drought stress without melatonin; D1+MT1: mild drought stress + 1 μmol·L^{-1} melatonin; D1+MT10: mild drought stress + 10 μmol·L^{-1} melatonin; D2-MT: moderate drought stress without melatonin; D2+MT1: moderate drought stress + 1 μmol·L^{-1} melatonin; D2+MT10: moderate drought stress + 10 μmol·L^{-1} melatonin. A: Root Surface Area; B: Root Volume; C: Root Fresh Weight; D: Root Dry Weight.

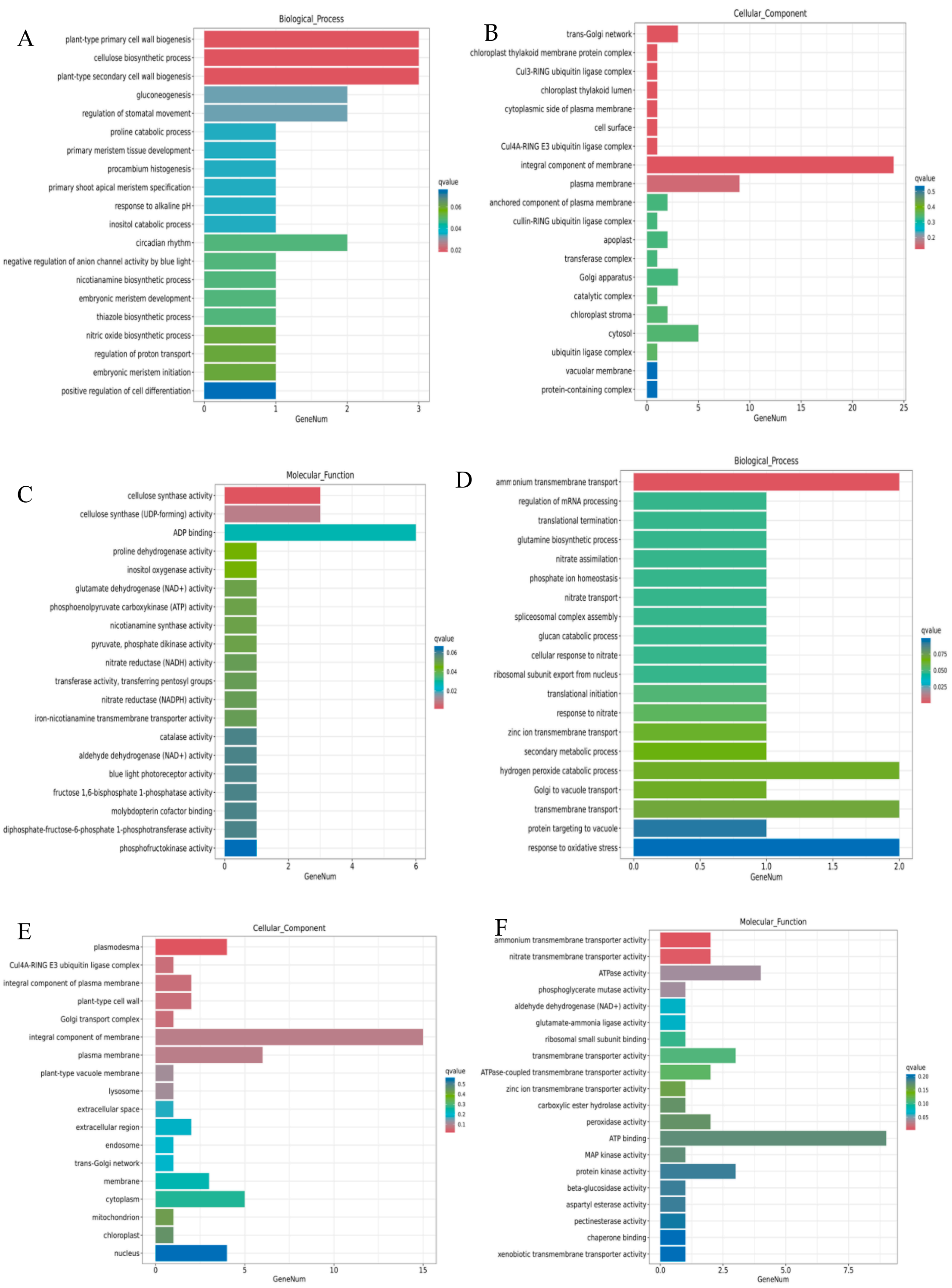

Figure S5. Analysis of functional enrichment of differentially expressed genes in sweet sorghum under drought stress with MT treatment.

A: Functional enrichment pathways of differentially expressed genes in the biological process (BP) category for the D1-MT vs D1+MT comparison; B: Functional enrichment pathways of DEGs in the cellular component (CC) category for the D1-MT vs D1+MT comparison; C: Functional enrichment pathways of DEGs in the molecular function (MF) category for the

D1-MT vs D1+MT comparison; D: Functional enrichment pathways of DEGs in the biological process (BP) category for the D2-MT vs D2+MT comparison; E: Functional enrichment pathways of DEGs in the cellular component (CC) category for the D2-MT vs D2+MT comparison; F: Functional enrichment pathways of DEGs in the molecular function (MF) category for the D1-MT vs D1+MT comparison. The color gradient of bars represents the q-value from the hyper geometric test, with lower q-value indicating higher statistical significance ($P < 0.05$).

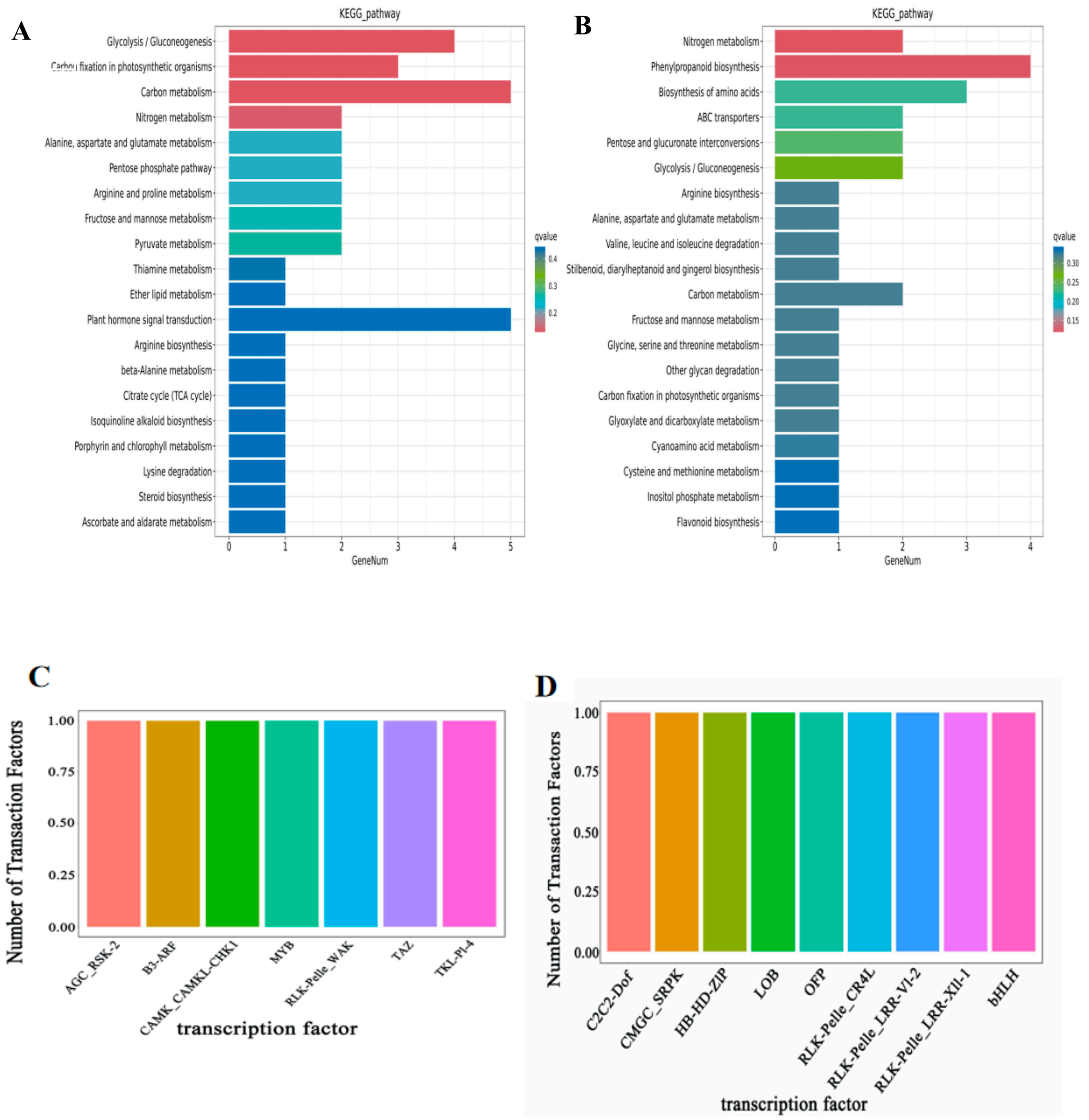

Figure S6. Analysis of metabolic pathways and transcription factors of differentially expressed genes in sweet sorghum under drought stress with MT treatment.

A: Enrichment results of differentially expressed genes (DEGs) in KEGG pathways for the D1-MT vs D1+MT comparison; B: Enrichment results of DEGs in KEGG pathways for the D2-MT vs D2+MT comparison; C: Seven transcription factor families associated with differentially expressed genes (DEGs) in the D1-MT vs D1+MT comparison; D: Nine transcription factor families associated with DEGs in the D2-MT vs D2+MT comparison. The color gradient of bars represents the q-value from the hyper geometric test, with lower q-value indicating higher statistical significance ($P < 0.05$).

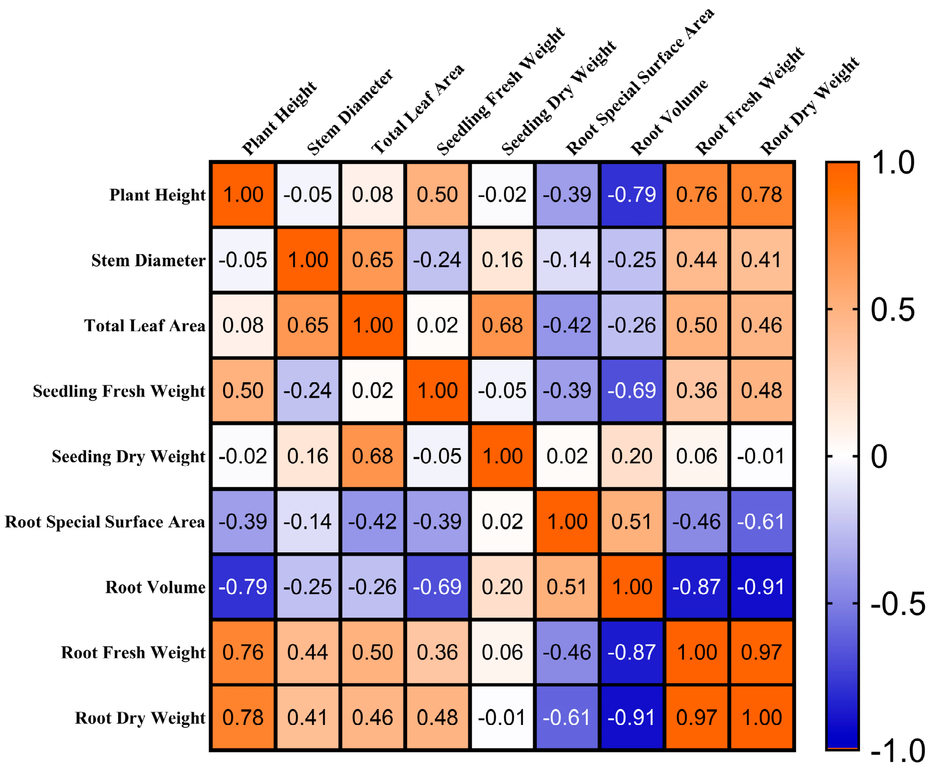

Figure S7. Correlation analysis among growth and root traits of sweet sorghum seedlings under different Treatments.

The figure shows Pearson correlation coefficients among nine traits: plant height, stem diameter, total leaf area, seedling fresh weight, seedling dry weight, root volume, root fresh weight, root dry weight, and root specific surface area. Orange represents positive correlations, and blue represents negative correlations.

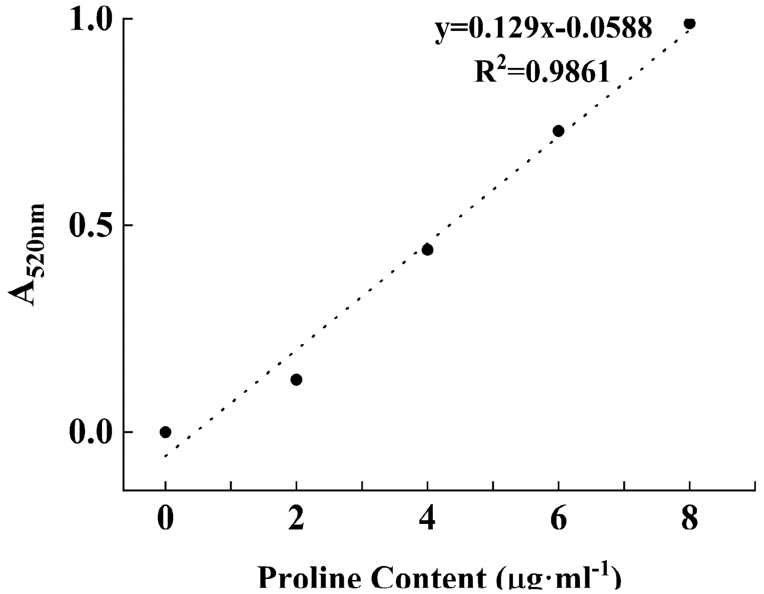

Figure S8. Standard curve for proline content determination.

The standard curve was plotted with proline concentration as the abscissa and absorbance at 520 nm as the ordinate. The linear regression equation was y = 0.129x – 0.0588, with a coefficient of determination R^2 = 0.9861. Each concentration point was measured in triplicate.

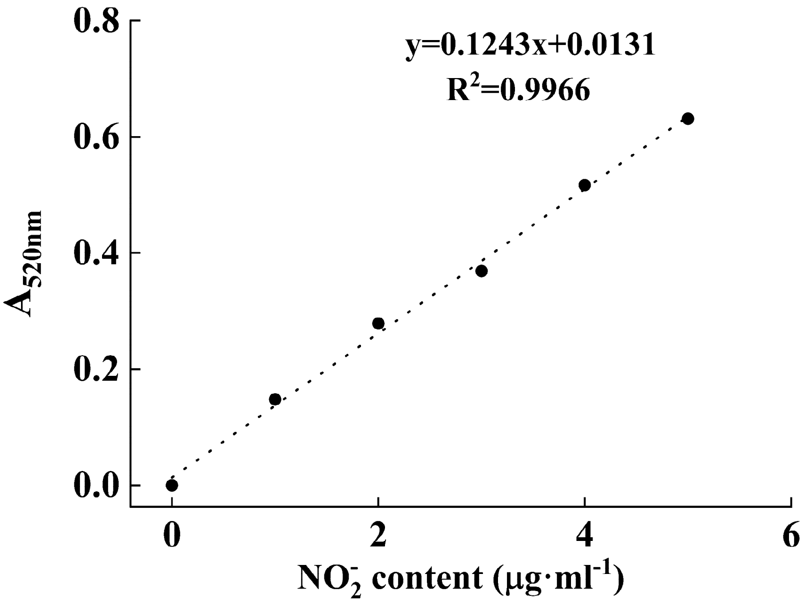

Figure S9. Standard curve for nitrate reductase activity determination using the sulfanilamide colorimetric method.

The standard curve was plotted with nitrite ion (NO_2^-) concentration ($\mu g \cdot mL^{-1}$) as the abscissa and absorbance at 520 nm as the ordinate. The linear regression equation was $y = 0.1243x + 0.0131$, with a coefficient of determination $R^2 = 0.9966$. Each concentration point was measured in triplicate. Nitrate reductase activity was calculated based on this standard curve.

Table S1. Sequencing data statistics table.

Samples	Clean reads	Clean bases	GC Content	%≥Q30
ck1-1	19,889,366	5,960,275,449	50.38%	97.16%
ck1-2	23,573,945	7,062,701,262	51.00%	95.68%
ck1-3	20,315,515	6,087,620,097	50.28%	96.96%
D1-2-1	21,625,597	6,467,470,933	50.47%	93.45%
D1-2-2	20,040,758	6,003,177,809	50.27%	97.09%
D1-2-3	23,686,270	7,097,299,105	49.77%	94.08%
D2-3-1	20,270,085	6,074,044,298	50.34%	97.27%
D2-3-2	20,108,174	6,026,763,019	49.97%	97.42%
D2-3-3	20,321,693	6,091,060,697	50.12%	98.04%
MT1 -4-1	20,070,535	6,014,087,851	49.89%	96.91%
MT1 -4-2	20,629,811	6,180,675,526	50.14%	96.89%
MT1 -4-3	20,658,857	6,190,263,935	49.78%	96.34%
MT2 -5-1	19,891,365	5,962,023,490	49.82%	97.19%
MT2 -5-2	21,120,971	6,328,715,926	49.89%	96.32%
MT2 -5-3	20,373,940	6,105,742,886	50.06%	97.40%

Note：Samples：Sample analysis number；ck：corresponds to 'control'；D1: corresponds to D1-MT in the text; D2: corresponds to D2-MT in the text; MT1: corresponds to D1+MT ; MT2: corresponds to D2+MT； Clean reads：Total number of paired-end reads in Clean Data; Clean bases：Total number of bases in Clean Data; GC content：The content of Clean Data GC; ≥Q30%：The percentage of bases in Clean Data with a quality score greater than or equal to 30.

Table S3. D1-MT vs D1+MT Gene pathways involved in photosynthesis.

KEGG participating pathway	Gene ID	Regulatory	Annotation
Carbon metabolism (ko01200)	SORBI_3004G011566	up	catalase isozyme 3 [Sorghum bicolor]

Table S4. D2-MT vs D2+MT Gene pathways involved in photosynthesis.

KEGG participating pathway	Gene ID	Regulatory	Annotation
Carbon fixation in photosynthetic organisms (ko00710); Carbon metabolism (ko01200)	SORBI_3006G157900	down	elicitor-responsive protein 3 [Sorghum bicolor]
Carbon metabolism (ko01200)	SORBI_3004G234100	down	uncharacterized protein LOC110434942 [Sorghum bicolor]
	SORBI_3006G157900	down	elicitor-responsive protein 3 [Sorghum bicolor]

Table S5. D1+MT vs D1+MT Gene pathways involved in osmoregulation.

KEGG participating pathway	Gene ID	Regulatory	Annotation
Arginine and proline metabolism (ko00330)	SORBI_3001G304700	up	proline dehydrogenase 2, mitochondrial [Sorghum bicolor]
	SORBI_3001G472000	down	polyamine oxidase-like [Sorghum bicolor]
Arginine biosynthesis (ko00220)	SORBI_3006G165400	up	glutamate dehydrogenase 2, mitochondrial isoform X1 [Sorghum bicolor]
Starch and sucrose metabolism (ko00500)	SORBI_3009G119200	up	hypothetical protein SORBI_3009G119200 [Sorghum bicolor]
Glycolysis / Gluconeogenesis (ko00010); Citrate cycle (TCA cycle) (ko00020); Carbon metabolism (ko01200)	SORBI_3001G326900	up	pyruvate, phosphate dikinase 2 isoform X1 [Sorghum bicolor]
	SORBI_3001G432800	up	phosphoenolpyruvate carboxykinase (ATP) [Sorghum bicolor]
	SORBI_3002G160300	down	pyrophosphate--fructose 6-phosphate 1-phosphotransferase subunit alpha isoform X3 [Sorghum bicolor]
	SORBI_3009G152700	up	fructose-1,6-bisphosphatase, cytosolic-like [Sorghum bicolor]

Table S6. D2-MT vs D2+MT Gene pathways involved in osmoregulation.

KEGG participating pathway	Gene ID	Regulatory	Annotation
Arginine biosynthesis (ko00220)	SORBI_3001G451500	down	glutamine synthetase root isozyme 1 [Sorghum bicolor]
Glycine, serine and threonine metabolism (ko00260)	SORBI_3004G234100	down	uncharacterized protein LOC110434942 [Sorghum bicolor]
Glycolysis / Gluconeogenesis (ko00010)	SORBI_3006G157900	down	elicitor-responsive protein 3 [Sorghum bicolor]

Table S7. D1-MT vs D1+MT Gene pathways related to nitrogen metabolism.

KEGG participating pathway	Gene ID	Regulatory	Annotation
Nitrogen metabolism (ko00910)	SORBI_3006G165400	up	glutamate dehydrogenase 2, mitochondrial isoform X1 [Sorghum bicolor]
	SORBI_3004G312500	up	nitrate reductase [NAD(P)H] [Sorghum bicolor]

Table S8. D2-MT vs D2+MT Gene pathways related to nitrogen metabolism.

KEGG participating pathway	Gene ID	Regulatory	Annotation
Nitrogen metabolism (ko00910)	SORBI_3004G009500	down	high-affinity nitrate transporter 2.1 [Sorghum bicolor]

Table S9. D1-MT vs D1+MT Antioxidation-related genes pathways.

KEGG participating pathway	Gene ID	Regulatory	Annotation
Ascorbate and aldarate metabolism(ko00053);Inositol phosphate metabolism (ko00562)	SORBI_3010G168800	up	probable inositol oxygenase [Sorghum bicolor]

Table S10. D2-MT vs D2+MT Antioxidation-related genes pathways.

KEGG participating pathway	Gene ID	Regulatory	Annotation
Phenylpropanoid biosynthesis (ko00940)	SORBI_3003G203600	down	aldehyde dehydrogenase family 2 member C4 [Sorghum bicolor]
	SORBI_3006G277500	up	peroxidase 12 [Sorghum bicolor]
	SORBI_3009G033300	down	peroxidase 5 [Sorghum bicolor]
	SORBI_3009G157300	down	uncharacterized protein LOC110430318 isoform X1 [Sorghum bicolor]
Flavonoid biosynthesis (ko00941)	SORBI_3006G211300	down	hypothetical protein SORBI_3006G211300 [Sorghum bicolor]
Inositol phosphate metabolism (ko00562)	SORBI_3006G157900	down	elicitor-responsive protein 3 [Sorghum bicolor]

Table S11. D1-MT vs D1+MT Gene pathways related to hormone signaling.

KEGG participating pathway	Gene ID	Regulatory	Annotation
Plant hormone signal transduction(ko04075)	SORBI_3002G042000	up	probable carboxylesterase 15 [Sorghum bicolor]
	SORBI_3003G302466	up	protein DOG1-like 3 [Sorghum bicolor]
	SORBI_3004G302200	up	uncharacterized protein LOC8078884 [Sorghum bicolor]
	SORBI_3010G073600	up	auxin response factor 16 isoform X1 [Sorghum bicolor]
MAPK signaling pathway - plant (ko04016)	SORBI_3004G016800	up	serine/threonine-protein kinase STY46 [Sorghum bicolor]

Table S12. D2-MT vs D2+MT Gene pathways related to hormone signaling.

KEGG participating pathway	Gene ID	Regulatory	Annotation
MAPK signaling pathway-plant (ko04016); Plant hormone signal transduction (ko04075)	SORBI_3001G278300	down	transcription factor bHLH19 [Sorghum bicolor]
